# Supplementary material for: BioFuse: an embedding fusion framework for biomedical foundation models
Source: PLoS One. 2026 Mar 18;21(3):e0320989. doi: 10.1371/journal.pone.0320989 (PMC12998865; doi:10.1371/journal.pone.0320989)
Supplement: S1 Appendix — (PDF) [file pone.0320989.s001.pdf]

S1 Appendix. BioFuse API

Typical workflow

BioFuse follows a five-stage pipeline:

1. Initialise `BioFuse` with a chosen set of foundation models.
2. Generate BioFuse embeddings for the training and validation splits.
3. Train and tune a downstream classifier on the fused embeddings.
4. Generate embeddings for the held-out test set with the same fusion module.
5. Evaluate the final classifier on the test embeddings.

High-level API

1. Initialise BioFuse

```
from biofuse import BioFuse

# List the available foundation models
print(BioFuse.get_supported_models())

# Create a BioFuse instance with the models you want
biofuse = BioFuse(models=['BioMedCLIP', 'UNI', 'rad-dino'])
```

2. Generate embeddings (train / validation)

```
train_emb, val_emb, fuse_model = biofuse.generate_embeddings(
        train_data,
        val_data,
        task=BioFuse.BINARY | BioFuse.MULTI_CLASS | BioFuse.MULTI_LABEL
)
```

3. Generate embeddings for new data

```
test_emb = fuse_model.generate_embeddings(test_data)
```

End-to-end example

```
from biofuse import BioFuse
from your_ml_lib import model_selection, tune_hp, evaluate, get_split

# 1.  Initialise with three backbones
biofuse = BioFuse(models=['BioMedCLIP', 'rad-dino', 'UNI'])
```

```

# 2.  Prepare data splits (images and labels)
train, val, test = get_split()

# 3.  Extract embeddings + fusion module
tr_emb, val_emb, fuse = biofuse.generate_embeddings(
        train, val, BioFuse.BINARY)

# 4.  Train and tune downstream classifier (e.g. XGBoost)
clf = model_selection(tr_emb, val_emb)
best = tune_hp(clf, tr_emb, val_emb)

# 5.  Embed test set and evaluate
te_emb = fuse.generate_embeddings(test)
metrics = evaluate(best, te_emb)

print(metrics)
```

Note: Code is illustrative; the final, fully documented API will be released in the public repository upon publication.
