# Supplementary material for: BioFuse: an embedding fusion framework for biomedical foundation models
Source: PLoS One. 2026 Mar 18;21(3):e0320989. doi: 10.1371/journal.pone.0320989 (PMC12998865; doi:10.1371/journal.pone.0320989)
Supplement: S2 Table — (PDF) [file pone.0320989.s002.pdf]

S2 Table. Hyperparameter Search Space for XG-Boost

Table 1: **XGBoost hyperparameters and their search ranges.** We employed Bayesian optimization via Weights & Biases to efficiently explore this search space across all BioFuse experiments on MedMNIST+. For each parameter, ranges were selected to encompass both conservative and aggressive values, enabling thorough exploration of model behavior across concatenation, self-attention fusion, and oracle-single configurations.

**Parameter**	**Range**	**Description**
learning_rate	[0.01, 0.03, 0.05, 0.1, 0.2, 0.3]	Step size shrinkage used to prevent overfitting. Lower values make the model more robust but slower to compute
n_estimators	[100, 250, 500, 750, 1000, 1500]	Number of boosting rounds or trees to be built
max_depth	[3, 6, 9, 12, 15]	Maximum depth of each tree. Larger values increase model complexity
max_leaves	[0, 31, 63, 127, 255]	Maximum number of leaves in each tree. 0 indicates no limit
min_child_weight	[1, 3, 5, 7]	Minimum sum of instance weight needed in a child node
subsample	[0.6, 0.8, 1.0]	Fraction of samples used for training each tree
colsample_bytree	[0.6, 0.8, 1.0]	Fraction of features used for training each tree
reg_alpha	[0, 0.1, 1, 10]	L1 regularization term on weights to prevent overfitting
reg_lambda	[0, 0.1, 1, 10]	L2 regularization term on weights to prevent overfitting
max_bin	[128, 256, 512]	Maximum number of discrete bins for continuous features
