# Supplementary material for: BioFuse: an embedding fusion framework for biomedical foundation models
Source: PLoS One. 2026 Mar 18;21(3):e0320989. doi: 10.1371/journal.pone.0320989 (PMC12998865; doi:10.1371/journal.pone.0320989)
Supplement: S3 Table — (PDF) [file pone.0320989.s003.pdf]

S3 Table. Optimal Hyperparameters for Concatenation Fusion

Table 1: **Optimal XGBoost hyperparameter configurations for concatenation fusion.** Dataset-specific configurations obtained through Bayesian optimization, showing how optimal depth, learning rate, and regularization vary across medical imaging tasks. These parameters were used for all concatenation-based experiments reported in the main text.

Dataset	max depth	colsample bytree	learning rate	max bin	max leaves	child weight	num estimators	reg alpha	reg lambda	sub sample
OrganAMNIST	12	0.8	0.3	128	127	7	750	10.0	0.1	0.8
OrganSMNIST	3	1.0	0.1	256	127	3	750	10.0	0.0	0.8
OrganCMNIST	15	1.0	0.1	128	255	5	1500	1.0	10.0	0.6
PathMNIST	15	1.0	0.2	256	255	1	1500	1.0	0.0	0.6
BloodMNIST	15	0.8	0.3	128	31	5	1000	10.0	10.0	1.0
OCTMNIST	15	0.8	0.3	128	31	3	1500	10.0	0.0	1.0
BreastMNIST	15	1.0	0.05	256	0	7	750	1.0	0.1	0.6
ChestMNIST	6	1.0	0.01	512	0	1	1000	10.0	10.0	1.0
TissueMNIST	9	0.6	0.2	512	127	1	1500	1.0	0.0	0.8
DermaMNIST	12	0.6	0.05	128	0	7	500	10.0	10.0	1.0
PneumoniaMNIST	15	0.8	0.3	128	63	1	100	0.1	10.0	1.0
RetinaMNIST	12	1.0	0.03	256	63	1	1000	1.0	0.1	0.8

Table 2: **Optimal XGBoost hyperparameter configurations for self-attention fusion.** These configurations demonstrate how the optimal parameters shift when using attention-based feature fusion compared to simple concatenation, with notable differences in tree depth and regularization across datasets.

**Dataset**	**max depth**	**colsample bytree**	**learning rate**	**max bin**	**max leaves**	**child weight**	**num estimators**	**reg alpha**	**reg lambda**	**sub sample**
OrganAMNIST	6	0.8	0.3	512	63	3	1500	0.1	1.0	0.8
OrganSMNIST	9	1.0	0.3	256	31	7	1500	0.1	0.1	1.0
OrganCMNIST	9	0.8	0.2	512	127	5	1500	10.0	0.1	1.0
PathMNIST	6	1.0	0.3	512	63	5	1500	0.0	1.0	1.0
BloodMNIST	15	1.0	0.3	512	127	5	1500	0.1	10.0	0.8
OCTMNIST	3	0.6	0.2	128	255	7	1500	10.0	0.0	1.0
BreastMNIST	12	0.6	0.2	512	127	7	250	0.1	10.0	0.8
ChestMNIST	3	1.0	0.01	256	127	1	1500	10.0	0.1	0.6
TissueMNIST	6	0.8	0.2	512	127	5	1500	0.1	1.0	0.6
DermaMNIST	15	1.0	0.1	128	0	5	1500	10.0	0.0	0.8
PneumoniaMNIST	3	1.0	0.2	128	127	5	1000	1.0	1.0	0.6
RetinaMNIST	12	0.8	0.2	256	127	3	1000	1.0	1.0	0.8
