# Supplementary material for: BioFuse: an embedding fusion framework for biomedical foundation models
Source: PLoS One. 2026 Mar 18;21(3):e0320989. doi: 10.1371/journal.pone.0320989 (PMC12998865; doi:10.1371/journal.pone.0320989)
Supplement: S4 Table — (PDF) [file pone.0320989.s004.pdf]

S4 Table. Optimal Hyperparameters for Self-Attention Fusion

Table 1: **Optimal XGBoost hyperparameter configurations for self-attention fusion.** These configurations demonstrate how the optimal parameters shift when using attention-based feature fusion compared to simple concatenation, with notable differences in tree depth and regularization across datasets.

Dataset	max depth	colsample bytree	learning rate	max bin	max leaves	child weight	num estimators	reg alpha	reg lambda	sub sample
OrganAMNIST	6	0.8	0.3	512	63	3	1500	0.1	1.0	0.8
OrganSMNIST	9	1.0	0.3	256	31	7	1500	0.1	0.1	1.0
OrganCMNIST	9	0.8	0.2	512	127	5	1500	10.0	0.1	1.0
PathMNIST	6	1.0	0.3	512	63	5	1500	0.0	1.0	1.0
BloodMNIST	15	1.0	0.3	512	127	5	1500	0.1	10.0	0.8
OCTMNIST	3	0.6	0.2	128	255	7	1500	10.0	0.0	1.0
BreastMNIST	12	0.6	0.2	512	127	7	250	0.1	10.0	0.8
ChestMNIST	3	1.0	0.01	256	127	1	1500	10.0	0.1	0.6
TissueMNIST	6	0.8	0.2	512	127	5	1500	0.1	1.0	0.6
DermaMNIST	15	1.0	0.1	128	0	5	1500	10.0	0.0	0.8
PneumoniaMNIST	3	1.0	0.2	128	127	5	1000	1.0	1.0	0.6
RetinaMNIST	12	0.8	0.2	256	127	3	1000	1.0	1.0	0.8
