# Supplementary material for: BioFuse: an embedding fusion framework for biomedical foundation models
Source: PLoS One. 2026 Mar 18;21(3):e0320989. doi: 10.1371/journal.pone.0320989 (PMC12998865; doi:10.1371/journal.pone.0320989)
Supplement: S5 Table — (PDF) [file pone.0320989.s005.pdf]

S5 Table. Optimal Hyperparameters for Oracle-Single Models

Table 1: **Optimal XGBoost hyperparameter configurations for oracle-single models.** These configurations represent the best parameters when using only the top-performing individual backbone for each dataset, serving as an upper bound baseline. The variation in optimal parameters across datasets highlights the importance of dataset-specific tuning.

**Dataset**	**max depth**	**colsample bytree**	**learning rate**	**max bin**	**max leaves**	**child weight**	**num estimators**	**reg alpha**	**reg lambda**	**sub sample**
BloodMNIST	15	1.0	0.10	128	0	5	1000	0.1	0.0	0.6
BreastMNIST	6	0.8	0.05	512	63	7	500	10.0	0.0	1.0
ChestMNIST	15	0.6	0.01	256	255	7	750	1.0	10.0	1.0
DermaMNIST	15	0.8	0.10	512	31	7	1500	10.0	10.0	1.0
OCTMNIST	9	0.6	0.30	128	0	3	1500	10.0	10.0	0.6
OrganAMNIST	3	0.8	0.10	256	63	5	1500	0.1	1.0	0.6
OrganCMNIST	15	1.0	0.01	256	127	1	250	1.0	10.0	0.8
OrganSMNIST	15	1.0	0.03	512	255	7	250	10.0	10.0	1.0
PathMNIST	12	0.6	0.20	256	31	5	1000	10.0	0.0	0.8
PneumoniaMNIST	12	0.8	0.01	256	127	7	750	1.0	0.0	1.0
RetinaMNIST	6	1.0	0.01	256	255	1	1500	1.0	10.0	1.0
TissueMNIST	6	0.6	0.20	512	0	3	1500	0.0	0.0	0.8
