# Supplementary material for: BioFuse: an embedding fusion framework for biomedical foundation models
Source: PLoS One. 2026 Mar 18;21(3):e0320989. doi: 10.1371/journal.pone.0320989 (PMC12998865; doi:10.1371/journal.pone.0320989)
Supplement: S6 Table — (PDF) [file pone.0320989.s006.pdf]

S6 Table. Computational Runtime Analysis

Table 1: **Computational runtime breakdown for each MedMNIST+ dataset using concatenation fusion.** Time measurements (in seconds) for embedding extraction, XGBoost model training, hyperparameter sweep, and total runtime across all datasets. ChestMNIST, TissueMNIST, PathMNIST, and OCTMNIST require the longest processing times, with total runtimes exceeding 15 hours. These measurements reflect the computational cost of processing medical images through nine foundation models and subsequent fusion training.

**Dataset**	**Embedding Extraction (s)**	**XGBoost Training (s)**	**Hyperparameter Sweep (s)**	**Total Runtime (s)**
BloodMNIST	1,746.17	1,417.82	7,322.63	10,486.62
BreastMNIST	38.80	881.68	446.41	1,366.89
ChestMNIST	10,942.26	21,169.70	146,870.49	178,982.45
DermaMNIST	1,049.82	1,237.15	5,874.42	8,161.39
OCTMNIST	11,173.09	3,064.42	42,990.72	57,228.23
OrganAMNIST	5,750.16	2,435.52	21,222.28	29,407.96
OrganCMNIST	2,326.41	1,576.48	14,499.64	18,402.53
OrganSMNIST	2,599.21	1,628.89	12,513.01	16,741.11
PathMNIST	10,294.71	3,347.86	41,134.15	54,776.72
PneumoniaMNIST	582.49	995.67	1,016.42	2,594.58
RetinaMNIST	160.01	825.31	1,894.00	2,879.32
TissueMNIST	23,992.92	5,055.03	105,523.04	134,570.99
