# Supplementary material for: BioFuse: an embedding fusion framework for biomedical foundation models
Source: PLoS One. 2026 Mar 18;21(3):e0320989. doi: 10.1371/journal.pone.0320989 (PMC12998865; doi:10.1371/journal.pone.0320989)
Supplement: S7 Fig — (PDF) [file pone.0320989.s007.pdf]

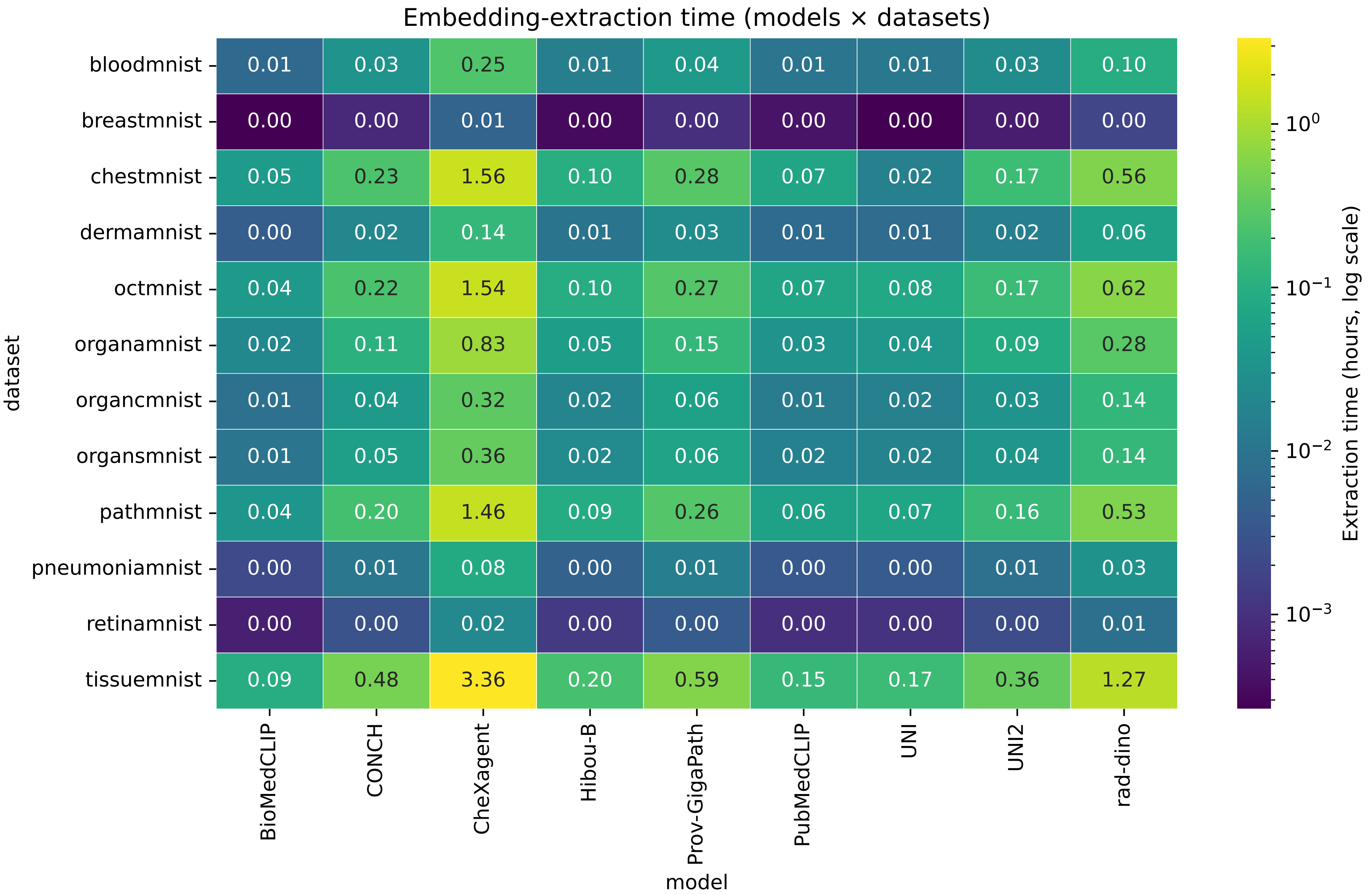

S7 Fig. Embedding extraction time across models and datasets.

Heat-map shows log-scaled hours required to obtain embeddings for each MedMNIST+ dataset from nine foundation models. CheXagent (third column) is roughly ten times slower than the other backbones, especially on ChestMNIST, OCTMNIST, PathMNIST, and TissueMNIST.
