# Supplementary material for: BioFuse: an embedding fusion framework for biomedical foundation models
Source: PLoS One. 2026 Mar 18;21(3):e0320989. doi: 10.1371/journal.pone.0320989 (PMC12998865; doi:10.1371/journal.pone.0320989)
Supplement: S8 Fig — (PDF) [file pone.0320989.s008.pdf]

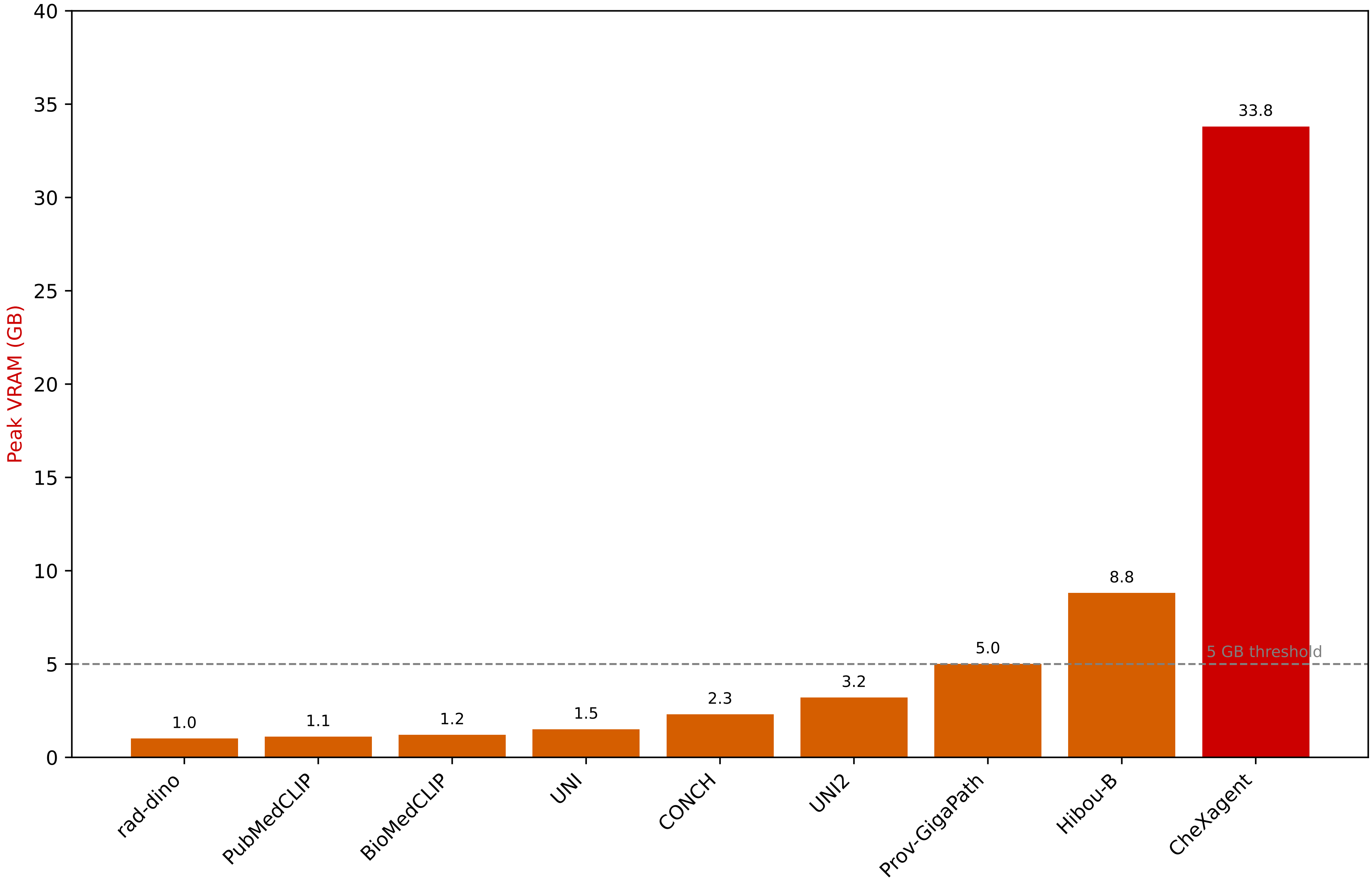

S8 Fig. Peak GPU VRAM usage (GB) for each model during embedding extraction

Memory requirements are consistent across datasets at steady state; values were measured on RetinaMNIST. CheXagent exceeds 33 GB, whereas most other backbones operate within a 5 GB envelope.
