# Supplementary material for: BioFuse: an embedding fusion framework for biomedical foundation models
Source: PLoS One. 2026 Mar 18;21(3):e0320989. doi: 10.1371/journal.pone.0320989 (PMC12998865; doi:10.1371/journal.pone.0320989)
Supplement: S9 Appendix — (PDF) [file pone.0320989.s009.pdf]

S9 Appendix. Foundation Model Pretraining Datasets

Table 1: **Pretraining datasets for biomedical foundation models used in BioFuse.** Details of training data sources for each model, including dataset scale, imaging modalities, and clinical contexts. These pretraining datasets have no overlap with MedMNIST+ evaluation benchmarks, ensuring independent assessment of model performance.

**Model**	**Dataset(s) Used**	**Dataset Details**
UNI	Mass-100K	Over 100M tissue patches from 100,426 WSIs covering 20 tissue types, sourced from MGH, BWH, and GTEx.
CONCH	Histopathology Image-Caption Pairs	1.17M pairs, combining educational resources and PubMed Central, limited to human histopathology.
Hibou-B	Proprietary WSI Dataset	1M WSIs from various tissue types, including 936,441 H&E and 202,464 non-H&E slides, and veterinary cases.
Prov-GigaPath	Prov-Path	1.3B 256×256 tiles from 171,189 pathology slides across 31 tissue types from 30,000+ patients.
CheXagent	CheXinstruct	6M image-instruction-answer triplets from 65 CXR datasets, including MIMIC-CXR, PadChest, and BIMCV-COVID-19.
RAD-DINO	Multi-CXR	838,000 CXR images from BRAX, MIMIC-CXR, NIH-CXR, PadChest, and private outpatient studies.
BioMedCLIP	PMC-15M	15M image-text pairs from 4.4M PubMed Central articles, covering 30 biomedical image types.
PubMedCLIP	ROCO	80,000 image-text pairs from PubMed, including ultrasound, X-ray, MRI, and other modalities across body regions.

MGH - Massachusetts General Hospital, BWH - Brigham and Women's Hospital, GTEx - Genotype-Tissue Expression Consortium
