# Supplementary material for: BioFuse: an embedding fusion framework for biomedical foundation models
Source: PLoS One. 2026 Mar 18;21(3):e0320989. doi: 10.1371/journal.pone.0320989 (PMC12998865; doi:10.1371/journal.pone.0320989)
Supplement: S10 Appendix — (PDF) [file pone.0320989.s010.pdf]

S10 Appendix. MedMNIST+ Dataset Characteristics and Independence Verification

Table 1: **MedMNIST+ evaluation datasets and their sources.** The MedMNIST+ collection comprises diverse labeled medical imaging datasets across diagnostic domains, each standardized to 224×224 RGB format. Source datasets, imaging modalities, and diagnostic categories are detailed below, with verification that none overlap with foundation model pretraining data, ensuring unbiased evaluation.

**Dataset**	**Source Dataset(s)**	**Description**
PathMNIST	Colorectal Cancer Histology Dataset	H&E-stained histology images from 86 slides, comprising over 100,000 patches, representing tissue types associated with colorectal cancer.
OCTMNIST	Retinal OCT Dataset	OCT images for retinal disease diagnosis, including macular degeneration and diabetic macular edema, from institutions like Shiley Eye Institute.
PneumoniaMNIST	Guangzhou Women and Children's Medical Center	5,232 pediatric chest X-ray images labeled to diagnose bacterial and viral pneumonia.
ChestMNIST	ChestX-ray8	Contains 108,948 frontal-view X-ray images of thoracic diseases such as pneumonia, cardiomegaly, and pneumothorax, derived from NIH.
DermaMNIST	HAM10000	10,015 dermatoscopic images from Queensland and Vienna clinics, covering seven skin lesion categories including melanoma and nevi.
BloodMNIST	Hospital Clinic of Barcelona Blood Cells	17,092 peripheral blood cell images, categorized into eight cell types, captured using the CellaVision DM96 analyzer.
BreastMNIST	Breast Ultrasound Dataset	780 breast ultrasound images from Baheya Hospital in Cairo, categorized as normal, benign, or malignant.
RetinaMNIST	DeepDRiD	Fundus images from 500 patients, used for grading diabetic retinopathy and image quality estimation.
TissueMNIST	Broad Bioimage Benchmark Collection (BBBC)	High-throughput microscopy images of cells and organisms for image-analysis benchmarking.
Organ{A,S,C}MNIST	Liver Tumor Segmentation Benchmark (LiTS)	CT images focused on liver tumors, collected from multiple institutions, labeled for primary and secondary tumor segmentation.
