# Supplementary material for: BioFuse: an embedding fusion framework for biomedical foundation models
Source: PLoS One. 2026 Mar 18;21(3):e0320989. doi: 10.1371/journal.pone.0320989 (PMC12998865; doi:10.1371/journal.pone.0320989)
Supplement: Attachment — Submitted filename: Response to Reviewers.pdf [file pone.0320989.s011.pdf]

We thank the reviewers for their constructive and insightful comments, which have substantially improved our manuscript. We have addressed all concerns as detailed below. Major revisions include: (1) comprehensive robustness evaluation using the MedMNIST-C corruption benchmark, (2) detailed computational efficiency analysis with runtime breakdowns, (3) two new ablation studies comparing fusion strategies, and (4) a new modular code repo with enhanced documentation. All line numbers refer to the new manuscript. Our replies in blue.

Response to Reviewer #1

(1) The manuscript only assessed the performance of BioFuse through AUC and accuracy, and does not involve other key indicators (such as computational efficiency, model robustness, and cross-dataset generalization ability). For example, the robustness of the model to noisy data or abnormal samples was not discussed. It is suggested to supplement multi-dimensional performance comparisons to comprehensively verify the practicability of the method.

1. **Robustness evaluation:** We have added a comprehensive robustness analysis using the MedMNIST-C benchmark, which applies 11 medically-motivated corruptions at 5 severity levels to all MedMNIST+ datasets. We report balanced error (BE) and relative balanced error (rBE) following the MedMNIST-C evaluation protocol (new section "Robustness to realistic image corruptions", lines 532, Table 7).

2. **Computational Efficiency:** We have substantially expanded our computational analysis. Due to a pipeline error discovered during revision (where 28×28 images were upscaled to 224×224 instead of using the native 224×224 MedMNIST+ images), we re-executed all experiments with corrected preprocessing. This provided an opportunity to collect detailed timing data across three phases: embedding extraction, model evaluation, and hyperparameter optimization. Results are presented in:

- Figure 6: End-to-end runtime visualization for all datasets
- S6 Table: Detailed per-dataset computational breakdown
- S7 Fig: Heatmap of embedding extraction times for each model-dataset pair

3. **Cross-dataset generalisation:** We investigated several public datasets (BUSI, ISIC, RSNA) for out-of-distribution evaluation but discovered that most are included in the pre-training corpora of our foundation models (detailed in S9 Appendix), rendering them unsuitable as independent test sets. We acknowledge this limitation and identify rigorous out-of-distribution evaluation as an important direction for future work (Limitations section, lines 693-).

(2) It is suggested to supplement and elaborate in the manuscript the possible feature conflict problems in multimodal feature fusion (such as the scale differences embedded in different models), and how to handle such problems.

We have added a new subsection "Feature-scale alignment" (lines 267) within the Fusion methodology section. This addition explains that: (i) vector concatenation preserves each model's features in isolated sub-blocks, avoiding cross-model normalization requirements, and (ii) our downstream classifier (XGBoost) makes splitting decisions based primarily on

relative feature ordering rather than absolute magnitudes, providing natural robustness to scale differences. We cite relevant literature [56,57,58] supporting this scale-invariance property of tree-based methods.

(3) The manuscript mentions the selection of model parameters, but does not explicitly list specific parameter values (such as the learning rate of XGBoost, maximum depth, etc.) and their impact on the results. It is suggested to provide a complete hyperparameter configuration table and discuss the parameter sensitivity analysis.

We now provide complete hyperparameter configurations in:

- S2 Table: XGBoost hyperparameter search ranges used for Bayesian optimization
- S3 Table: Optimal parameters for concatenation fusion (main results)
- S4 Table: Optimal parameters for self-attention fusion ablation
- S5 Table: Optimal parameters for oracle-single ablation

Each table lists all nine XGBoost hyperparameters for each of the 12 datasets. Regarding sensitivity analysis, we note that exhaustive parameter sensitivity sweeps across nine hyperparameters, 12 datasets, and three fusion variants would require thousands of additional training cycles, exceeding our computational resources. Consistent with established MedMNIST+ benchmarking practices, we report optimal configurations discovered through Bayesian optimization.

(4) It is suggested that ablation experiments should be added to the manuscript to verify the independent contribution of key designs in BioFuse (such as vector join strategies and automatic model selection) to performance, quantify the improvement ratio of each part to the final result, and compare it with other fusion methods.

We have added two comprehensive ablation studies:

1. Self-attention fusion vs. Concatenation (Table 5, lines 482-): This ablation replaces simple concatenation with a learnable multi-head self-attention mechanism.
2. Oracle-single vs. Concatenation (Table 6, lines 517-): This ablation compares fusion against using only the single best model per dataset.

Both ablations include detailed discussions of the findings and their implications for the fusion strategy.

Response to Reviewer #2

(1) To enhance the study, consider expanding the evaluation to include segmentation, object detection, and regression tasks, as well as testing on real-world clinical datasets.

We appreciate this suggestion for broadening BioFuse's evaluation. As noted in our response to Reviewer #1 (Comment 1.3), we investigated several clinical datasets but found that most are included in our foundation models' pre-training corpora (S9 Appendix) or serve as sources for MedMNIST+ (S10 Appendix). Regarding task diversity, we acknowledge that extending to segmentation and object detection represents an important future direction. Our Limitations section (lines 692-) to explicitly identify these as priorities for future work, while

noting that such extensions would require substantial architectural modifications beyond the current scope.

(2) Additionally, incorporating a more diverse set of foundation models from various biomedical domains and exploring alternative fusion strategies, such as attention-based methods, could further improve the framework's versatility and efficiency.

We have addressed both aspects of this comment:

1. Alternative Fusion Strategy: We implemented and evaluated a self-attention fusion mechanism as requested (Table 5, lines 482-). This learnable approach underperformed simple concatenation on most datasets, providing empirical validation for our design choice.

2. Model Diversity: Our current pool of nine foundation models already spans diverse biomedical domains (histopathology, radiology, multimodal vision-language) and architectures. We discuss future incorporation of MRI, ultrasound, genomics, and clinical text models in the Limitations section (lines 699-). The framework's modular architecture facilitates easy integration of new models as they become available.

(3) Addressing computational efficiency and enhancing interpretability through explainability techniques would also be valuable.

Computational Efficiency: Please see our detailed response to Reviewer #1 (Comment 1.2). We have added comprehensive runtime analysis including wall-clock measurements across all computational phases (Figure 6, S6 Table, S7 Figure) and GPU memory requirements (S8 Figure). We discuss this in depth in the "Computational considerations" section (lines 654-).

Interpretability: We acknowledge interpretability as a critical requirement for clinical deployment and discuss this in the limiations section (lines 706-) and identify the integration of saliency maps, feature attribution methods, and attention visualization as essential future work for clinical acceptance and regulatory approval.

(4) Finally, ensuring the models are representative and unbiased, along with providing comprehensive documentation and tutorials, would make BioFuse more accessible and impactful for a broader audience.

We have taken several steps to improve BioFuse's accessibility:

1. Enhanced Architecture & Documentation: We are actively developing improved documentation and code architecture. A new modular version of the codebase (https://github.com/mnhcorp/biofuse/tree/v1.0) is currently in progress and will include:

- A modular pluggable architecture
- Detailed architecture documentation
- Installation guides
- API reference documentation
- Example notebooks demonstrating common use cases

While this enhanced version is still under development, the current release provides functional code for reproducing all experiments. Additionally, we have made pre-computed embedding caches available on Zenodo to eliminate computational barriers for users.

2. Bias and Representativeness: We acknowledge the importance of model bias assessment. While our nine foundation models were trained on diverse, large-scale datasets (S9 Appendix), systematic bias evaluation across demographic groups remains future work. We have added this consideration to our Limitations section (lines 700-).
