# Supplementary material for: BioFuse: an embedding fusion framework for biomedical foundation models
Source: PLoS One. 2026 Mar 18;21(3):e0320989. doi: 10.1371/journal.pone.0320989 (PMC12998865; doi:10.1371/journal.pone.0320989)
Supplement: Attachment — Submitted filename: Response_to_Reviewers_auresp_2.pdf [file pone.0320989.s012.pdf]

Dear Editors of PLOS ONE,

Thank you for your message and for the continued handling of our manuscript, **“BioFuse: an embedding fusion framework for biomedical foundation models”** (PONE-D-25-10720). We appreciate the Academic Editor’s guidance on improving the manuscript’s structure prior to acceptance. We have implemented the requested changes and believe the revised version is now substantially clearer and easier to navigate.

Major revisions include:

1. **Improved hierarchical organization and numbering:** Added a consistent sequential numbering system across headings and subheadings (e.g., 1, 1.1, 1.1.1) to improve readability and cross-referencing.
2. **Merged “Background” and “Related work”:** Integrated these into a single section titled **“Background and related work”**, improving logical flow from general context to prior literature and the specific gap addressed.
3. **Integrated BioFuse into Methods:** Moved the BioFuse description into the Methods section as **“BioFuse framework”**, aligning the system description with the rest of the methodological details.
4. **Consolidated experimental methodology for clarity:** Combined the former “Experimental design” and “Experimental procedure” into a single **“Experiments”** section with a clearer internal hierarchy. We also updated the **Objectives** subsection to explicitly enumerate the full experimental scope, including robustness and ablation analyses, consistent with the Results section.
5. **Consistency and cross-references:** Updated internal references throughout to match the revised section structure.

Please do not hesitate to contact us if any additional information is required.

Kind Regards,

Mirza Nasir Hossain
University of St Andrews
Email: mnh3@st-andrews.ac.uk
